# Two-Step Iterative Medical Microwave Tomography

**DOI:** 10.3390/s24216897

**Published:** 2024-10-27

**Authors:** Zekun Zhang, Heng Liu, Xiang Gao, Zeyu Zhang, Zhongxia Simon He, Luoyuan Liu, Rui Zong, Zhizhen Qin

**Affiliations:** 1 School of Information and Electronics, Beijing Institute of Technology, Beijing 100081, China; 3220215110@bit.edu.cn (Z.Z.); 7520230190@bit.edu.cn (Z.Z.); 2 School of Cyberspace Science and Technology, Beijing Institute of Technology, Beijing 100081, Chinazhongxia@bit.edu.cn (Z.S.H.); 3 Chalmers Industriteknik, 412 58 Göteborg, Sweden; 4 Sydney Smart Technology College, Northeastern University at Qinhuangdao, Qinhuangdao 066004, China; 5 Department of Neurosurgery, The First Medical Center of PLA General Hospital, Beijing 100853, China

**Keywords:** signal processing, microwave tomography, inverse scattering, Born approximation

## Abstract

In the field of medical imaging, microwave tomography (MWT) is based on the scattering and absorption characteristics of different tissues to microwaves and can reconstruct the electromagnetic property distribution of biological tissues non-invasively and without ionizing radiation. However, due to the inherently nonlinear and ill-posed characteristics of MWT calculations, actual imaging is prone to overfitting or artifacts. To address this, this paper proposes a two-step iterative imaging approach for rapid medical microwave tomography. This method establishes corresponding objective functions for microwave imaging across multiple frequencies and conducts iterative calculations on images at varying resolutions. This effectively enhances image clarity and accuracy while alleviating the issue of prolonged computational time associated with imaging complex structures at high resolution due to insufficient prior information during iterative processes. In the electromagnetic simulation section, we simulated a three-layer brain model and conducted imaging experiments. The results demonstrate that the algorithm significantly enhances imaging resolution, accurately pinpointing cerebral hemorrhages at different locations using an eight-antenna array and successfully reconstructs tomography images with a hemorrhage area radius of 1 cm. Lastly, experiments were conducted using a medical microwave tomography platform and four simplified human brain models, achieving millimeter-level accuracy in MWT.

## 1. Introduction

With the rapid advancement of medical imaging technology, traditional methods such as X-ray tomography (CT), magnetic resonance imaging (MRI), and ultrasound imaging have become widely used in clinical diagnosis. However, these techniques present certain limitations, including the radiation risk associated with X-rays [1], the high cost of MRI [2], and the dependence of ultrasound imaging on image quality [3]. In light of these challenges, microwave tomography (MWT) has emerged as a promising medical imaging technology due to its distinct advantages [4].

MWT operates based on the scattering and absorption properties of tissues when exposed to microwaves, enabling the non-invasive reconstruction of the electromagnetic properties of biological tissues without the need for ionizing radiation. It offers benefits such as low risk, non-ionizing radiation, and cost-effectiveness in medical imaging [5]. MWT has proven particularly advantageous in soft tissue imaging and shows great potential for detecting brain conditions like strokes and tumors [6].

Additionally, advances in materials science and embedded manufacturing technology have facilitated the development of miniaturized and wearable devices for MWT [7]. Compared to traditional imaging techniques like CT and MRI, MWT not only provides high-contrast images of soft tissues but also differentiates the electrical properties of tissues, such as variations in conductivity and the dielectric constant. This capability plays a critical role in the early detection of abnormal tissues, offering a significant advantage in medical diagnostics.

In terms of microwave inverse scattering imaging algorithms, there are mainly confocal imaging algorithms and scattered field iterative methods. Confocal microwave imaging (CMI) uses the reflection principle of electromagnetic waves between geometric objects for imaging [8,9,10]. It is fast but only has good imaging effects on simple structures and has poor imaging effects on complex structures. The iterative methods transform the inverse scattering problem into an optimization problem by approximating the scattering field [11] and obtaining the optimal solution for imaging by iterating the scattering area.

By convolving the scattering model with Green’s function to obtain forward and backward scattered fields close to the analytical solution of the Helmholtz equation, the iterative algorithm performs computational imaging. This includes techniques such as Born approximation iteration (BIM) [12,13], contrast source inversion (CSI) [14,15], coupled dipole [16], and the subspace optimization method [17], among others. Within the BIM framework, the above various iterative methods can be combined, such as the magnetic field fluctuation contrast source operator embedded in the improved BIM [18], the use of level sets to determine the search direction based on the reconstruction of the shape and position of the object with a known contrast profile [19], or the use of Jacobian matrices or approximations of the derivatives of the residual function to optimize the iteration [20], and various distorted Born Iterative Methods (DBIM) have been proposed to improve imaging quality and speed [21,22,23].

In the iterative algorithm, sufficient prior information is very important in ensuring imaging efficiency and quality. By incorporating prior information, such as the contrast of the target area tissue, the final imaging quality can be significantly improved. For example, spatial distribution [24] and dielectric constant values [25,26,27] can be used as prior information. Compared with the traditional Born iterative algorithm, the Quadratic programming Born Iterative Method (QPBIM) uses quadratic programming optimization and prior information on upper and lower bounds of contrast values to solve the inverse problem and has better stability and noise immunity [28]. These methods yield good imaging results for small targets at high resolution. However, increasing the resolution significantly extends the iteration time, and the absence of sufficient prior information can introduce artifacts in small target detection, impairing accurate target recognition.

For the above problems, this paper proposes a two-step iterative scheme that optimizes the scattered field errors using an antenna array to reconstruct tomography images. The approach combines the Born Iterative Method with quadratic programming for a more effective two-step iteration process. The preliminary iteration has a lower resolution and is used to quickly and roughly calculate the dielectric constant in the imaging area as the prior information for the second iteration, reducing the imaging time required for high-resolution iteration. The second iteration improves the resolution through interpolation and further iterates on image edges and abnormal areas by constructing a mask, thereby achieving fast iterative imaging at high resolution. At the same time, the second iteration shields the coupling medium in the imaging area, minimizing artifacts outside the target region and providing a clearer visualization of the measured target’s structure. During the imaging process, we considered the electromagnetic characteristics of the human tissue. We added constraints on the range of relative permittivity and conductivity within the imaging area, thereby effectively reducing the number of iterations while improving stability. In the experimental part, we constructed both simulation and model experiments to analyze and verify the theoretical performance of the algorithm and the imaging ability in the actual environment. The experimental verification shows that the algorithm can not only accurately locate the hematoma position in the multi-layer brain model, but also has strong anti-noise ability and can accurately image in the actual environment.

## 2. Methods

**Figure 1:**
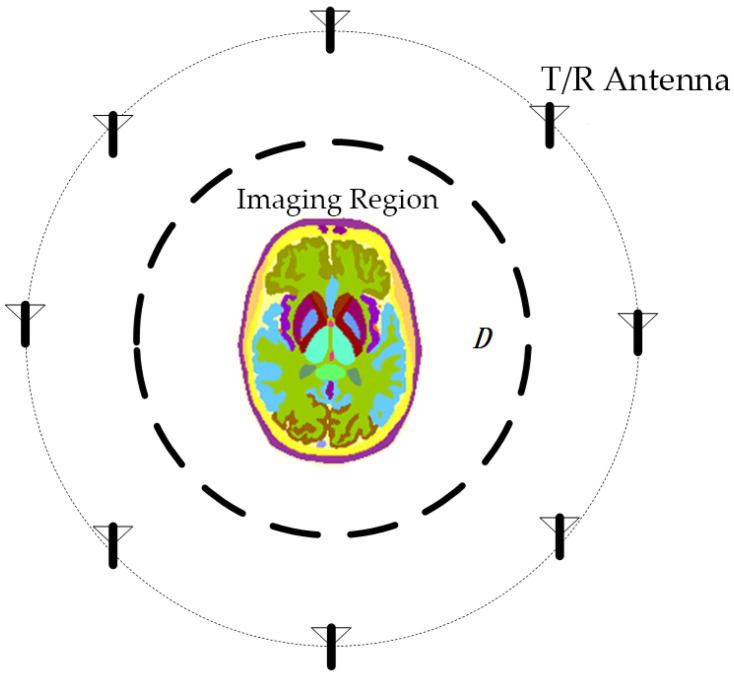
Schematic diagram of microwave tomography. The brain is used as an imaging target, and antennas arranged around it can transmit and receive microwave signals.

MWT is a technique that utilizes microwave irradiation to capture internal information and reconstruct tomography images. As microwaves propagate through the human tissue, electromagnetic scattering varies at sites with differing permittivity. Solving the inverse scattering problem is essential to ascertain the dielectric properties of each tissue for MWT. Specifically, as illustrated in Figure 1, the antenna array surrounds the imaging area D, and the dielectric constant distribution within the imaging area D can be determined based on the incident field and the total field received at the antenna. This enables accurate localization of the lesion.

The equation for the total field $E_{r}^{t}$ at location r in electromagnetic scattering problems is as follows:

(1) $$E_{r}^{t}=E_{r}^{i}+E_{r}^{s},$$

(2) $$E_{r}^{s}={k_{b}}^{2}\int G_{r,r^{\prime }}\chi_{r^{\prime }}E_{r^{\prime }}^{t}dr^{\prime },$$

where r refers to the position of observation point, $r^{\prime }$ refers to the position of source point in the integration domain, $E_{r}^{i}$ is the incident field, $E_{r}^{s}$ is the scattering field, G is Green’s function, $k_{b}$ is the wavenumber and χ is the difference in the complex dielectric constant between the presence and absence of the scatterer, given by the following:

(3) $$\chi =\frac{\varepsilon_{r}}{{\varepsilon_{r}}_{b}}-1-\frac{j\left(\sigma -\sigma_{b}\right)}{\omega \varepsilon_{0}{\varepsilon_{r}}_{b}}.$$

The dyadic Green’s function for homogeneous medium in 2-D problems is given in terms of a Hankel function of the second kind as follows:

(4) $$G_{r,r^{\prime }}=-\frac{jk_{b}^{2}H_{0}^{\left(2\right)}\;\left(k_{b}\left|r-r^{\prime }\right|\right)}{4}.$$

The inverse scattering problem in MWT is inherently nonlinear and ill-posed. This is due to the disparity between the large number of unknowns (points in the imaging region) and the relatively limited number of known quantities (receiving antennas). As a result, the solution to the inverse scattering problem in MWT is not unique.

The Born Iterative Method is commonly employed for solving inverse scattering problems. This method begins with an initial estimate of the complex permittivity within the imaging region, followed by sequential solutions for the total electric field and the complex permittivity difference χ. In each iteration, the forward electromagnetic scattering problem is resolved to determine the total electric field. Subsequently, the inverse scattering problem is tackled to derive the complex permittivity difference χ, continuing until the iteration stopping condition is met or the maximum number of iterations is reached. The distribution of permittivity within the imaging region is then inferred from the calculated complex permittivity difference, χ, in the final iteration.

This paper addresses the inverse problem using the Born iterative optimization error method and introduces a two-step iterative method to configure imaging parameters for different regions. Initially, a priori information is acquired from the imaging area through rapid iterations at low resolution. Subsequently, image resolution is enhanced, and a second iteration focuses on the abnormal area and tissue edges. This method reduces imaging time while enhancing the clarity of the results.

### 2.1. Error Estimation

In order to solve the distribution of complex permittivity in the imaging region, the imaging region D is discretized and divided into a grid of small uniform squares. Adopting the *δ* (Dirac delta function) as the basis function, the integration in Equation (2) can be approximated in the form of summation with the following expression:

(5) $$E_{r}^{s}=\sum_{r^{\prime }}^{R}g_{r,r^{\prime }}\chi_{r^{\prime }}E_{r^{\prime }}^{t}\;.$$

And the specific expression of $g_{r,r^{\prime }}$ in the discretization of the dyadic Green’s function is given by the following:

(6) $$g_{r,r^{\prime }}=-\frac{j\pi k_{b}\sqrt{\frac{dxdy}{\pi }}J_{1}(k_{b}\sqrt{\frac{dxdy}{\pi }})H_{0}^{\left(2\right)}\;\left(k_{b}\left|r-r^{\prime }\right|\right)}{2}.$$

In the formula, dxdy is the unit area after discretization of the imaging region, $J_{1}$ is the Bessel function of the first kind, and $H_{0}^{\left(2\right)}$ is the second class of zeroth-order Hankel functions.

The inverse electromagnetic scattering problem can be described as an optimization problem, that is, taking χ as the independent variable, calculating the error between the measured value and the estimated value.

(7) $${Loss}_{base}=\sum_{n}^{N-1}{\left|E_{n}^{s}-\sum_{r^{\prime }}^{R}g_{r,r^{\prime }}\chi_{r^{\prime }}E_{r^{\prime }}^{t}\right|}^{2}.$$

The formula above represents the error function that quantifies the difference between the measured value and the estimated value of the scattered field. Here, n refers to the receiving antenna, and N−1 represents the number of all antennas excluding the current transmitting antenna. The scattered field $E_{n}^{s}$ is obtained by subtracting the incident field from the total measured field at the receiving antenna. The specific form of the error optimization problem above is shown below:

(8) $$\min{Loss}_{base}=\underset{\chi }{\min}\sum_{n}^{N-1}{\left|E_{n}^{s}-\sum_{r^{\prime }}^{R}g_{r,r^{\prime }}\chi_{r^{\prime }}E_{r^{\prime }}^{t}\right|}^{2}.$$

When the ${Loss}_{base}$ is approximately zero, the estimated value of the scattered field is equal to the measured value of the scattered field, and χ is the difference in complex permittivity within the imaging region.

Considering all N transmitting antennas and F frequency points, using m to refer to the transmitting antenna and f to refer to the frequency, the total error function ${Loss}_{scatter}$ can be expressed as follows:

(9) $${Loss}_{scatter}=\sum_{m}^{N}\sum_{f}^{F}\sum_{n}^{N-1}{\left|E_{n}^{s}-\sum_{r^{\prime }}^{R}g_{r,r^{\prime }}\chi_{r^{\prime }}E_{r^{\prime }}^{t}\right|}^{2}.$$

In MWT’s inverse scattering problem, the number of variables far surpasses the number of provided linear equations. Directly solving the optimization problem can result in the non-uniqueness of solutions and lead to overfitting. To address this issue, we incorporated regularization terms into the objective function to enhance solution stability and mitigate overfitting risks.

In order to ensure the imaging quality, we add the values of all pixels to the error function. This helps prevent any single point with an excessively large value from causing the solution to get stuck in a local optimum. The error function of the pixel value ${Loss}_{pixel}$ is as follows at this time:

(10) $${Loss}_{pixel}=\alpha_{1}\sum_{r}^{R}\left|u_{r}\right|+\alpha_{2}\sum_{r}^{R}\left|v_{r}\right|,$$

where $\alpha_{1}$ and $\alpha_{2}$ are regularization coefficients, and they can affect the final imaging result. $u_{r}$ is the real part of the product of the dielectric constant and the actual area at position r, and $v_{r}$ is the imaginary part of the product. By increasing the value of α, the final generated result can have a lower average value.

To enhance the smoothness and continuity of the imaging results, we incorporate the image gradient into the objective function expression. The gradient is approximated by the ratio of the difference between adjacent pixels in both horizontal and vertical directions to the actual distance difference between these pixels. The error term ${Loss}_{gradient}$ is expressed as follows:

(11) $${Loss}_{gradient}=\beta_{1}\sum_{r}^{R}{\left|w_{r}\right|}^{2}+\beta_{2}\sum_{r}^{R}{\left|m_{r}\right|}^{2}=\beta \sum_{r}^{R}\left({\left|w_{r}\right|}^{2}+{\left|m_{r}\right|}^{2}\right),$$

where $w_{r}$ is the gradient of the pixel at position r in the horizontal direction, and $m_{r}$ is the gradient of the pixel in the vertical direction. We sum the approximated gradients and include them as an additional term in the objective function. In practical imaging scenarios, distinguishing between horizontal and vertical imaging effects is unnecessary. Therefore, we consolidate $\beta_{1}$ and $\beta_{2}$ in the formula into a single parameter β. The error function ${Loss}_{1}$ is derived to optimize the imaging process and is represented as follows:

(12) $${Loss}_{1}={Loss}_{scatter}+{Loss}_{pixel}+{Loss}_{gradient}.$$

By limiting the value ranges of $u_{r}$, $v_{r}$, $w_{r}$, and $m_{r}$, the stability of the imaging results can be effectively improved during the imaging calculation process. In the process of preliminary iteration, we use lower resolution for fast iteration and use the iteration result as the initial state of the second iteration.

### 2.2. Two-Step Iteration

In the preliminary iterative process, we use low image resolution to quickly obtain results, and use the results as basic prior information to introduce into the second step to improve the efficiency of high-resolution imaging. Because the image resolution and the β parameter in the error function limit the gradient of the entire imaging area, and due to the presence of the coupling medium, the imaging result at this time will have slight edge blurring. This makes it hard to accurately distinguish the location of areas in multiple layers, such as the skull, skin, and brain. In order to improve the image resolution and subdivide different structures, we further perform a second iterative imaging of edge locations within the image.

To more clearly observe the changes in the target during the iterative process, we can mark the largest connected region within the imaging area that has values similar to the coupling medium as the background. During the output process, this region is set to zero in the resulting image. This approach helps remove the background medium from the imaging results while also reducing the impact of external artifacts.

At this time, we only iteratively optimize the areas in the image that belong to the edge of the medium. Since there are sudden changes in the electrical properties of the medium during the process, we remove the error term about the gradient during the second iteration of the optimization process to obtain a new error function ${Loss}_{2}$, the expression is written as follows:

(13) $${Loss}_{2}=\sum_{m}^{N}\sum_{f}^{F}\sum_{n}^{N-1}{\left|E_{n}^{s}-\sum_{r^{\prime }}^{R}g_{r,r^{\prime }}\chi_{r^{\prime }}E_{r^{\prime }}^{t}\right|}^{2}+\alpha_{1}\sum_{r}^{R}\left|u_{r}\right|+\alpha_{2}\sum_{r}^{R}\left|v_{r}\right|.$$

This expression has a similar form to the previous expression, but when optimizing it as an objective function, the current iteration result needs to be masked using Mask to exclude pixel values in non-edge areas. Mask is an array composed of 0 and 1 with the same size as the original image:

(14) $$Mask=\left\{\begin{array}{c} 0,\;where\;\forall \left|d\right|\in \left[0,d_{0}\right]:{\left|w_{r+d}\right|}^{2}+{\left|m_{r+d}\right|}^{2}<\tau \\ 1,\;where\;\exists \left|d\right|\in \left[0,d_{0}\right]:{\left|w_{r+d}\right|}^{2}+{\left|m_{r+d}\right|}^{2}\geq \tau \end{array}\right..$$

We use the gradient value at each pixel position as the criterion for identifying edge areas. In the formula, τ is the decision threshold. The equation signifies that if a pixel within a distance $d_{0}$ from a specific position exceeds the threshold τ, the corresponding position in the array is set to 1.

(15) $$\tau =\underset{r}{\mathrm{ave}}\left({\left|w_{r}\right|}^{2}+{\left|m_{r}\right|}^{2}\right).$$

In the subsequent application process, we adopt the square value of the average gradient of the image as the decision threshold and set $d_{0}$ to 1 cm.

## 3. Simulation Experiments and Results

**Figure 2:**
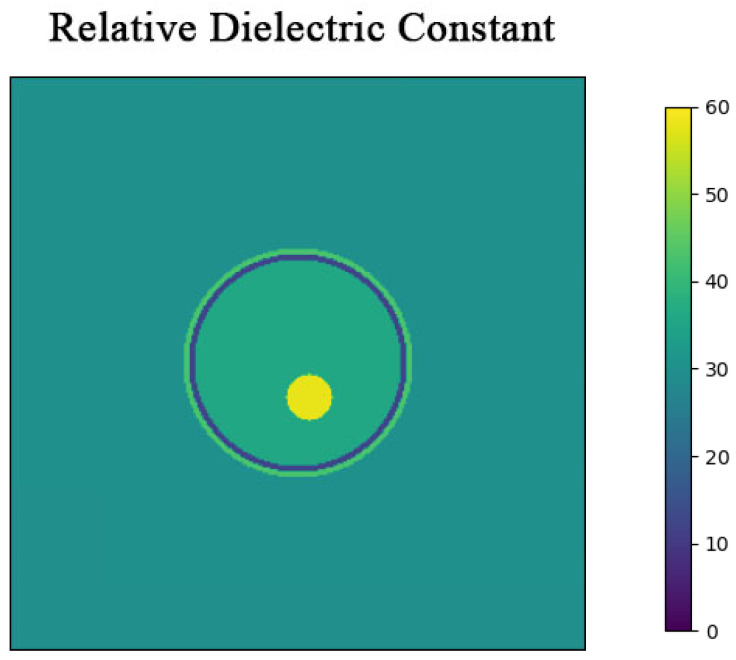
Three-layer simulation model cross-section. The model is a cylinder with a height of 15 cm, surrounded by a coupling medium with a relative permittivity of 30.

To evaluate the performance of the Born Iterative Method based on error optimization, a simplified three-layer brain model with a radius of 10 cm was developed. The human brain comprises various tissues, including brain tissue, skull, and skin. When microwaves irradiate the brain, they traverse these tissues sequentially. Therefore, in simulations, the human brain model is often simplified into a skull model with multiple nested layers. The three-layer human brain model used in this study, from the outermost to the innermost, includes the following: a skin layer, a skull layer, and a brain layer. Figure 2 illustrates the nested structure of the three-layer brain model. The complex relative permittivity $\varepsilon_{r}$ of the brain layer is calculated using the Fourth-order Debye model [29].

(16) $$\varepsilon_{r}\left(\omega \right)=\varepsilon_{\infty }+\sum_{i=1}^{4}\frac{∆\varepsilon_{i}}{1+j\omega \tau_{i}}+\frac{\sigma_{s}}{j\omega \varepsilon_{0}}.$$

In the model, $\varepsilon_{\infty }$ is the dielectric constant at infinite frequency, $∆\varepsilon_{i}$ is the change in dielectric constant related to the i-th Debye relaxation process, $\tau_{i}$ is the relaxation time constant for the i-th process, and $\sigma_{s}$ is the static conductivity. The dielectric constant difference $∆\varepsilon_{i}$ and time constant $\tau_{i}$ are obtained through an optimization process based on a genetic algorithm (GA).

**Figure 3:**
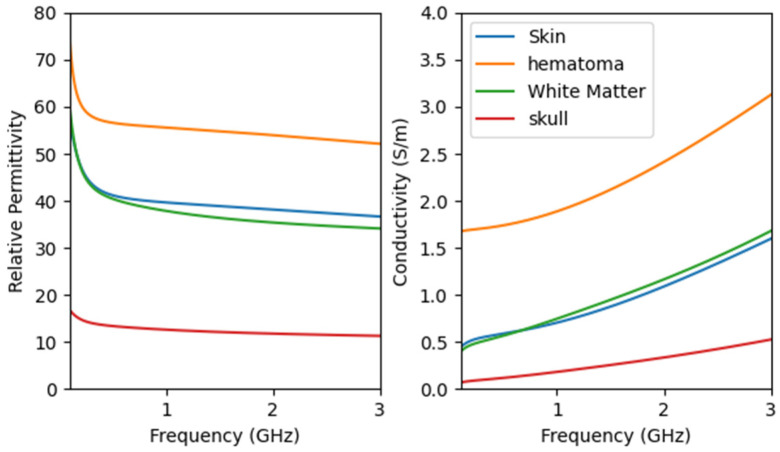
Properties of head tissues with frequency using the derived Fourth-order Debye model.

The specific values of the electromagnetic properties of each tissue in the human brain model are shown in Figure 3. The microwave frequency band used spans from 0.5 GHz to 2 GHz, covering a total bandwidth of 1.5 GHz, with 201 points sampled at equal intervals. Too high a frequency will decay quickly in organic media, resulting in excessive noise that affects imaging performance.

Based on the electromagnetic properties of the model, once electromagnetic waves penetrate the skin tissue, their signal strength exponentially decays due to electrical conductivity, mirroring real-world testing conditions. Following the construction of the brain model, eight antennas are evenly positioned 24 cm from the brain’s center. The setup involves multiple transmission and reception cycles, where each antenna sequentially emits TM waves in the z-direction while the remaining antennas receive them.

### 3.1. Parameter Optimization

As the regularization parameters vary, the iterative reconstruction of images is affected accordingly. Achieving accurate values for two-step iterative imaging necessitates suitable preliminary iteration outcomes. In this section, we will investigate different α and β to determine optimal defaults for brain imaging, and select 10 frequency points at equal intervals as calculation parameters.

In the scenario where the brain model includes a bleeding area centered at coordinates (1, −3) cm with a radius of 2 cm, adjusting the values of α and β impacts the imaging results as follows:

Different values of α or β can significantly affect the imaging results. From Table 1, it is evident that varying the parameters α and β—whether they are excessively large or small—degrades the quality of image reconstruction.

When α or β is too small, the image exhibits unevenness, leading to pronounced overfitting artifacts.

An excessively large α primarily influences the error function by diminishing pixel values excessively, resulting in a dark and indistinct overall image.

A high β restricts the range of pixel changes significantly, causing abnormal areas like blood clots to appear faint and nearly indistinguishable while smoothing the image edges excessively, thereby blurring the image.

When α and β are selected as $5\times 10^{3}$ and $5\times 10^{-6}$, respectively, the imaging effect is the most stable under the current experimental environment.

### 3.2. Simulated Imaging Results

In the previous section, we performed a parameterized exploration of different values of α and β. We set the values of $\alpha_{1}$ and $\alpha_{2}$ to $5\times 10^{3}$, and the value of β to $5\times 10^{-6}$ for microwave imaging.

**Figure 4:**
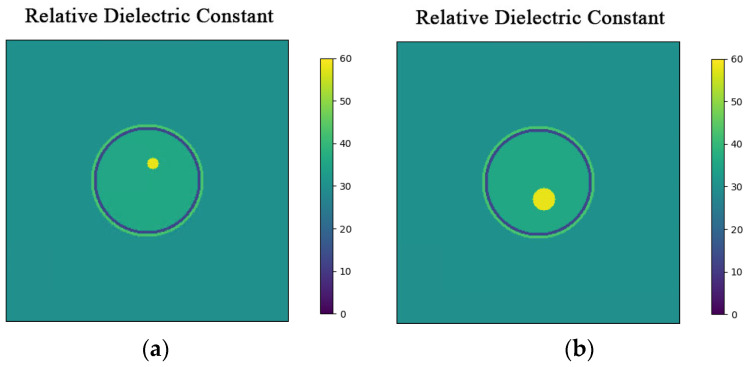
Hematomas in different locations. (**a**) Brain model with a hematoma at (1, 3) with a radius of 1 cm; (**b**) Brain model with a hematoma at (1, −3) with a radius of 2 cm.

In this section, the three-layer brain model is imaged with two different configurations: one includes a circular bleeding area centered at coordinates (1, 3) with a radius of 1 cm, and the other includes a circular bleeding area centered at coordinates (1, −3) with a radius of 2 cm. The original structure of the model is shown in Figure 4.

**Figure 5:**
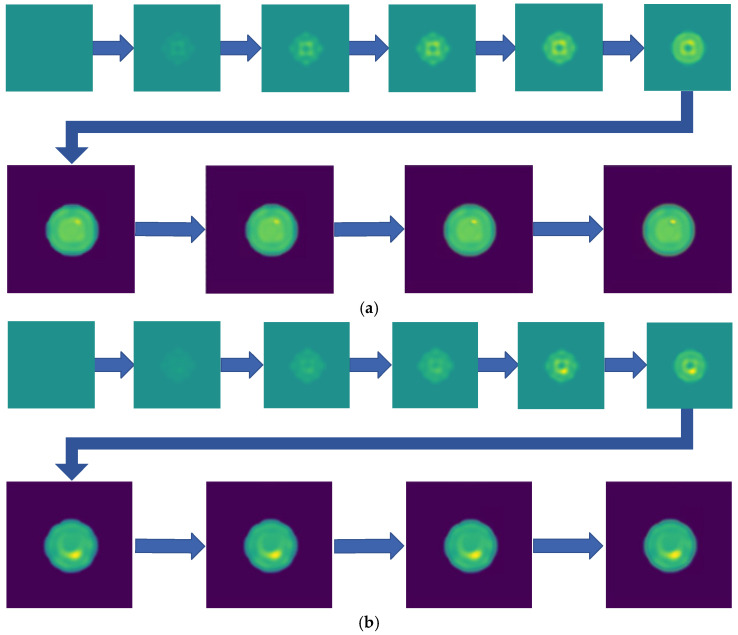
Imaging results with hematoma. (**a**) Imaging result of a hematoma at (1, 3); (**b**) Imaging result of a hematoma at (1, −3). The first two rows are the preliminary iteration part of 100 × 100 resolution, and the second row is the process of using the mask array to perform a second iteration on the edge position after increasing the resolution to 200 × 200.

Two-step imaging iterations are conducted on the two simulation models, and the results are depicted in Figure 5. The first row displays the preliminary iterations using a 100 × 100 low resolution, while the second row illustrates the process of applying a mask array for a second iteration on the edge positions after increasing the resolution to 200 × 200.

As shown in Figure 5, compared to a simple initial iteration, the two-step iteration process significantly improves the accuracy of tissue edge detection and the clarity of the medium by applying a mask and eliminating external media. It accurately distinguishes the multi-layer structures at the simulation region edges, revealing previously obscured details. The internal bleeding area is also notably enhanced, becoming more distinct compared to the initial imaging iteration.

By recording the error between the scattered field and relative permittivity throughout the iteration process and comparing it to the true values, we can plot the curves of the relative error $\xi_{E}$ for the scattered field and the error $\xi_{\varepsilon }$ for the relative permittivity, respectively.

**Figure 6:**
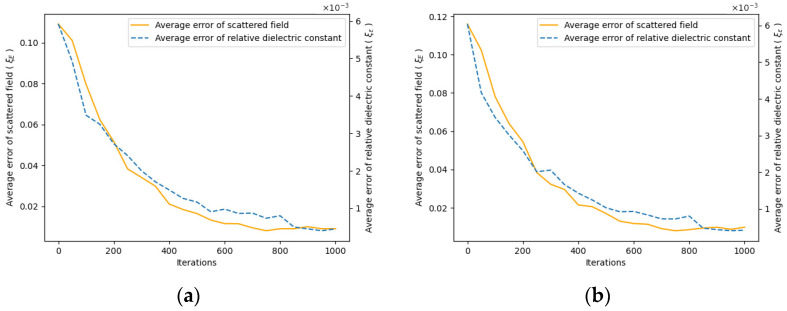
Iteration error curve. As the iteration proceeds, the error between the measured scattered field and the estimated scattered field gradually decreases, and the dielectric constant distribution approaches the true value. (**a**) Error curve for the hematoma at (1, 3); (**b**) Error curve for the hematoma at (1, −3).

The initial 800 iterations focus on low-resolution imaging. As shown in Figure 6, following the second iteration, there is a noticeable reduction in relative permittivity error, highlighting the effectiveness of the two-step iteration algorithm in significantly minimizing imaging errors, enhancing image resolution, and capturing additional details. In terms of time efficiency, this approach saves more than half the time compared to directly employing high-resolution imaging.

## 4. Model Experiments and Results

Based on the aforementioned simulation experiments, we constructed microwave detection equipment and an experimental platform. In this chapter, we developed two distinct brain models to approximate normal and hemorrhagic conditions using imaging solutions. Subsequently, we employed the proposed method to conduct inverse imaging.

### 4.1. Experimental Detection Platform

**Figure 7:**
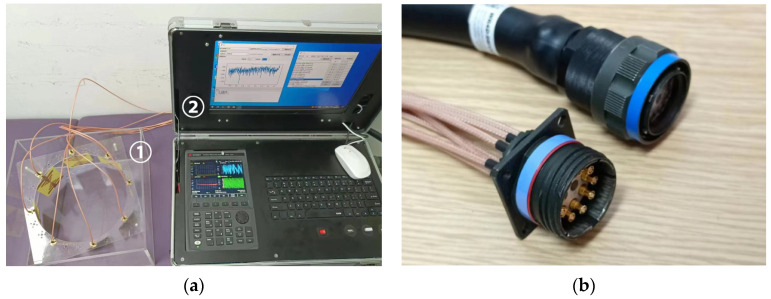
Experimental microwave detection equipment and cables. The two components are interconnected via a radio frequency cable bundle during detection. (**a**) Detection equipment (1) and control equipment (2); (**b**) Cable bundle connecting the equipment.

The experimental platform consists front-end detection equipment and back-end control equipment. The front-end detection setup includes antennas, a coupling medium composed of glycerin, and its container. The back-end control equipment consists of a vector network analyzer and associated RF control components, as shown in Figure 7.

The back end is equipped with a lithium battery to power the entire device, and it switches different transmitting and receiving antennas in sequence to complete the task during detection. Although the equipment utilizes identical materials and components across different channels, each channel of the cable bundle still requires calibration before the experiment to mitigate errors stemming from variations between cables. An automated calibration program was developed for this project. By manually switching the connections of the electronic calibration kit to different ports, the calibration status for each port combination is recorded and can be recalled during actual testing.

**Figure 8:**
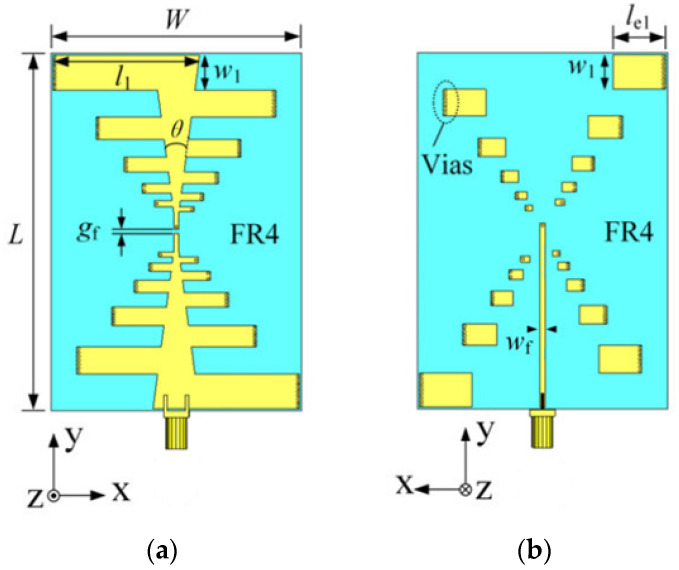
The proposed low-profile ultrawideband antenna with unidirectional pattern. (**a**) Front side of the antenna; (**b**) Back side of the antenna. (L = 91 mm, W= 64 mm, H = 1 mm, $H_{s}$ = 3 mm, $l_{1}$ = 37.4, $w_{1}$ = 8.7 mm, $l_{e1}$ = 13.5 mm, $w_{f}$ = 1.25 mm, $g_{f}$ = 1 mm, θ = 15°).

The antenna utilized at the front end is an omnidirectional ultra-wideband log-periodic antenna loaded with a solution and constructed with an FR4 substrate material, enabling effective transmission and reception within a coupling medium of high dielectric constant [30]. Consistent with the previous simulation tests, we employed eight antennas for transmitting and receiving RF signals. Figure 8 shows the geometry of the radiating element, which is a double-layer folded log-periodic structure for bandwidth enhancement and size miniaturization, with Vias used to interconnect the layers and achieve the folded configuration.

Using water, cornstarch, gelatin, and sodium chloride as raw materials, we can prepare dielectric solutions with various dielectric constants [31]. The configuration and composition ratio of each simulated tissue is based on the information provided in Table 2.

**Figure 9:**
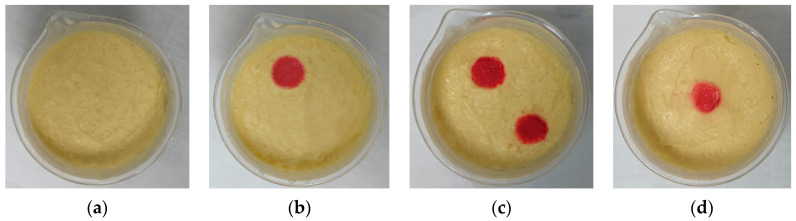
Experimental brain models. (**a**) Brain model with no hemorrhage; (**b**) Hemorrhagic brain model with a hemorrhage area in the upper left corner; (**c**) Hemorrhagic brain model with two hemorrhage areas in the upper left and lower right corners; (**d**) Hemorrhagic brain model with a hemorrhage area at the center.

Figure 9 shows the created brain models A–D. The model tested in the experiment consists of two parts: yellow represents the normal area of cerebral white matter, and red represents the abnormal bleeding tissue. Model A represents a normal brain, and B, C, and D are hemorrhage models. In model B, a 2 cm diameter cylindrical simulated hemorrhage area is placed in the upper left corner. In model C, an additional similar simulated hemorrhage lesion is placed in the lower right corner, resulting in two bleeding areas to be detected. Model D places a hemorrhage area at the center.

**Figure 10:**
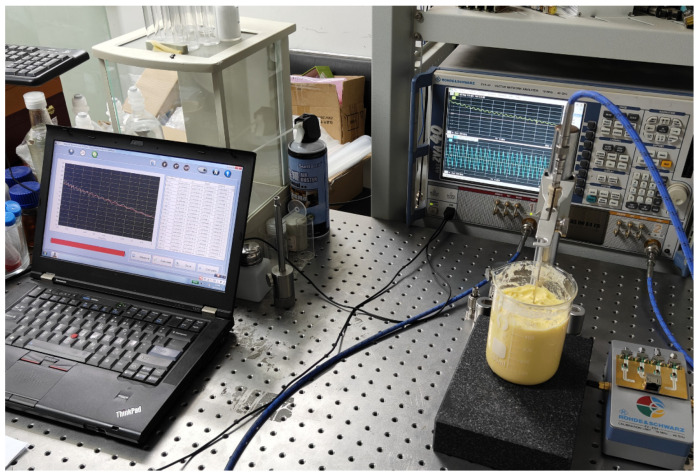
Dielectric constant measurement of the experimental phantom. The test was conducted using a ZVA40 PNA, and the dielectric constant was measured with a MEMF-C1203 probe.

**Figure 11:**
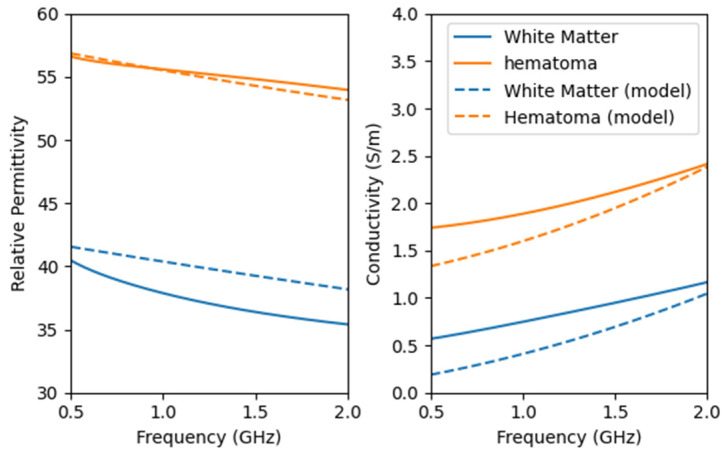
Dielectric properties of the configured brain model and real head tissues. The dashed line represents the properties of the configured model.

We used the dielectric constant test fixture, as shown in Figure 10, to evaluate the configured brain model. Figure 11 presents the dielectric constant and conductivity curves for both the configured model and real tissue.

The configured model exhibits a high dielectric similarity to real tissue. The addition of NaCl effectively enhances the conductivity of the model, bringing it closer to the properties of real hematoma.

**Figure 12:**
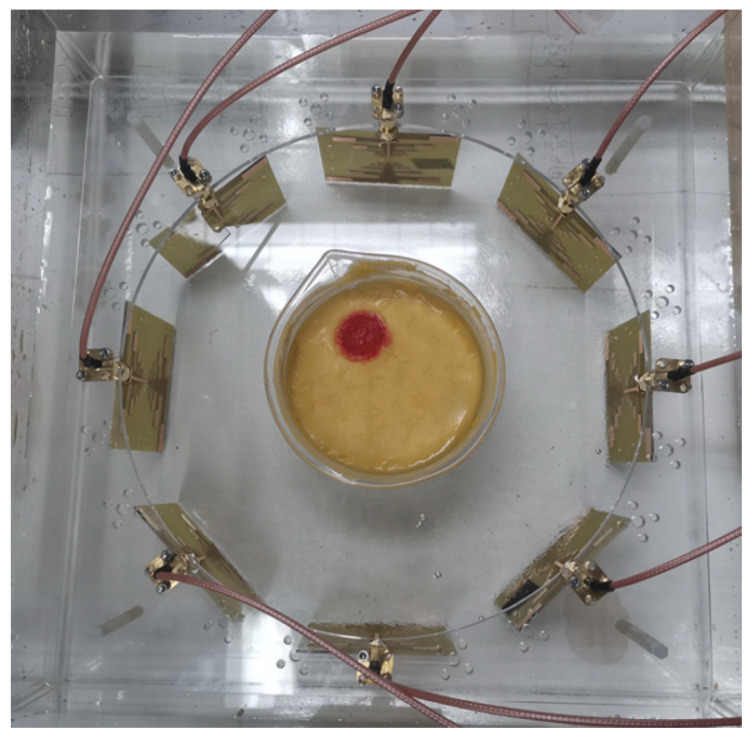
Detection equipment and experimental brain model. Both the antennas and the model are immersed in a coupling medium solution to minimize the effects caused by sudden changes in the dielectric constant. The antennas in the array operate sequentially to collect the scattered signals generated by the model. By subtracting the background field, the influence of antenna coupling is effectively reduced.

In order to collect the scattered field generated by the model, tests need to be conducted separately on the setup containing only the coupling medium and the setup with the model included. During the test, the control device switches the active antenna in Figure 12 using the RF switch array. It then retrieves the calibration parameters for the currently selected channel, waits for the data to stabilize, and records the corresponding scattering parameters as detection data.

### 4.2. Experimental Imaging Results

**Figure 13:**
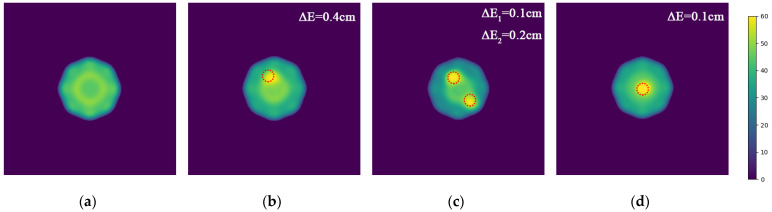
Imaging results of the model experiment with the MWT platform. (**a**) Result of normal brain model A; (**b**) Result of brain model B with a hemorrhage area in the upper left corner; (**c**) Result of brain model C with two hemorrhage areas in the upper left and lower right corners; (**d**) Result of brain model D with a hemorrhage area at the center.

After detection and imaging iterations, the final imaging result is shown in Figure 13. We simulate the distance between the actual position of the hemorrhage $\left(x_{A},{\;y}_{A}\right)$ and the imaging position $\left(x_{B},{\;y}_{B}\right)$ as the imaging error ΔE of the hemorrhage brain model.

(17) $$∆E=\sqrt{{\left(x_{A}-x_{B}\right)}^{2}+{\left(y_{A}-y_{B}\right)}^{2}}.$$

Since both the antenna and the test target are immersed in a coupling medium solution, the majority of external electromagnetic interference entering the device from the air is reflected by the coupling medium. This effectively mitigates noise interference in the imaging results and can be ignored.

The results demonstrate that the interior of the normal model A appears relatively uniform, whereas the bleeding area can be accurately identified in models B, C, and D. Compared to the actual model, the error ΔE of the lesion center is less than 1 cm. Due to the thinness of the glass container holding the model, it is not prominently visible in the imaging results.

Based on the actual imaging results, it is evident that the proposed microwave imaging method effectively facilitates the imaging of multi-layered brain models. It accurately detects the precise location of stroke hemorrhage areas with millimeter-level accuracy.

## 5. Discussion

To achieve precise MWT, this paper proposes a two-step iterative imaging algorithm based on an optimization method. The imaging capability and noise resistance of the algorithm under different parameters are analyzed through simulation experiments. Finally, the effectiveness of the algorithm is verified through model experiments by building a measurement platform and creating four simplified human brain models.

The main contributions of this article are as follows: (1) proposing a scheme for dynamically modifying the imaging resolution and objective function for multi-step iteration, addressing the issue of long iterative calculation times due to the lack of prior information for complex structures in MWT; (2) utilizing a mask to cover non-interest areas in high-resolution images, which reduces computational resource consumption and enhances tissue edge clarity in the second iteration. Additionally, by removing external media from the imaging results, the influence of the coupling medium on the images is further minimized; (3) developing an integrated detection platform and validating the algorithm through model experiments using this medical MWT platform.

The proposed method considers the dielectric constant characteristics of human tissue, sets the corresponding objective function for microwave imaging at multiple frequencies, and performs two-step iterative calculations on images of different resolutions. This approach effectively addresses the problem of long calculation times for high-resolution imaging of complex structures. In the initial iteration, we reduce complexity by lowering the image resolution to quickly obtain prior information. After increasing the resolution, we optimize the second iteration by adding a coverage mask to reduce the amount of gradient value calculation.

In the simulation experiment, this paper used an eight-antenna array to preliminarily verify the algorithm and analyze the influence of algorithm parameters on imaging results. Compared to the original iterative algorithm, this method quickly delivers multi-layer structure imaging results with clearer edges and higher resolution. The algorithm can accurately locate intracranial hematomas with a radius of 1 cm.

In the model experiment, we created two different models—normal and hemorrhagic—and utilized the MWT platform to detect and image these models. The platform used the same eight-antenna array as the simulation part. The experimental results demonstrate that the microwave imaging algorithm proposed in this paper, combined with the experimental platform, effectively images the cranial models. It accurately distinguishes and locates bleeding symptoms, achieving millimeter-level precision imaging. Furthermore, the presence of the coupling medium solution effectively reflects and mitigates external noise when entering the equipment.

In future studies, we aim to validate and refine the algorithm further by integrating both model experiments and clinical examples. We plan to incorporate artificial intelligence to explore MWT methods combined with time-frequency domain imaging schemes. Adequate prior information is crucial for optimizing the imaging outcomes with our optimization algorithm. During actual tests, the uncertainty regarding the size and position of the detected target can lead to overfitting issues in the iterative process, causing significant deviations between imaging results and the actual conditions. Therefore, it is necessary to further optimize the structure of the antenna array and the shape of the field line. Additionally, obtaining sufficient prior information through a blend of time and frequency domains presents an effective approach to enhancing iterative calculations in the frequency domain.

## Figures and Tables

**Table 1:** Imaging results under different parameters.

	$\alpha =5\times 10^{2}$	$\alpha =5\times 10^{3}$	$\alpha =5\times 10^{4}$
$\beta =5\times 10^{-7}$	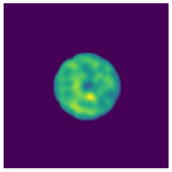	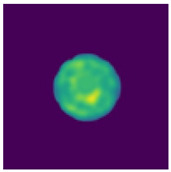	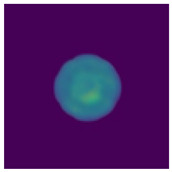
$\beta =5\times 10^{-6}$	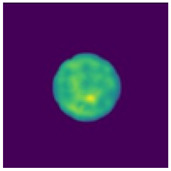	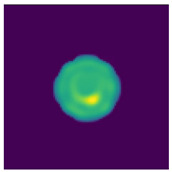	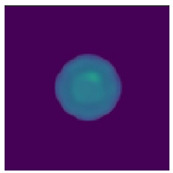
$\beta =5\times 10^{-5}$	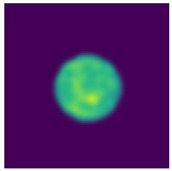	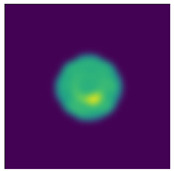	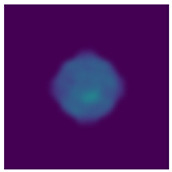

**Table 2:** Ingredient of the experimental phantom.

Medium Type	Water (mL)	Corn Starch (gm)	Gelatin (gm)	NaCl (gm)	Glycerin (mL)
White matter	364	185	9.8	0	0
Hematoma	100	14	20	1.2	0
Coupling medium	20	0	0	0	80

## Data Availability

Data are available at reasonable request from the corresponding author.
